# Controlling droplet splashing and bouncing by dielectrowetting

**DOI:** 10.1038/s41598-021-00771-z

**Published:** 2021-11-01

**Authors:** M. A. Quetzeri-Santiago, J. R. Castrejón-Pita, A. A. Castrejón-Pita

**Affiliations:** 1 Department of Engineering Science, University of Oxford, Oxford, OX1 3PJ UK; 2 School of Engineering and Materials Science, Queen Mary University of London, London, E1 4NS UK; 3 Present Address: Mesoscale Chemical Systems Group, MESA+ Institute and Faculty of Science and Technology, University of Twente, P.O. Box 217, 7500AE Enschede, The Netherlands

**Keywords:** Applied physics, Fluid dynamics

## Abstract

Stopping droplets from bouncing or splashing after impacting a surface is fundamental in preventing cross-contamination, and the spreading of germs and harmful substances. Here we demonstrate that dielectrowetting can be applied to actively control the dynamics of droplet impact. Moreover, we demonstrate that dielectrowetting can be used to prevent droplet bouncing and suppress splashing. In our experiments, the dielectrowetting effect is produced on a flat substrate by two thin interdigitated electrodes connected to an alternating current potential. Our findings show that the strength of the electric potential can affect the dynamic contact angle and regulate the spreading, splashing and receding dynamics at the right time-scales.

## Introduction

Surface wettability is crucial to the performance of several industrial applications, such as spray coating, inkjet printing, microfluidic methods and airplane icing^1–4^. In practice, the wetting properties of solid substrates are often modified through three methods: by chemical processes such as a corona plasma treatment^5–7^ or by the addition of fluorocarbon containing coatings; by changing the surface roughness^8^; and through the use of electric fields^3,9,10^. The first two methods permanently, or semi-permanently, change the wettability of a surface, but electrowetting and dielectrowetting methods offer mechanisms that alter it on demand^11^. In a typical electrowetting setup, a conductive sessile droplet rests on a flat conductive surface (an electrode) coated with a dielectric layer, and a second electrode, often a metallic needle, is introduced at the top of the droplet^9^. The wettability of the substrate, as quantified by the contact angle, is then controlled by an electric potential applied between the needle and the flat electrode. In this scenario, wettability changes are the result of the interaction between free ions on the droplet and the polarised solid dielectric layer^9^. Electrowetting shows a wide control over the contact angle and the droplet contact surface. However, electrowetting methods do not achieve complete wetting (or hydrophilicity) as they present wetting saturation and show contact angle hysteresis^12^. Furthermore, traditional electrowetting configurations require that one of the electrodes remains in permanent contact with the droplet^3^. This restriction makes electrowetting unsuitable for some applications, such as technologies based on droplet impact^12,13^. In contrast, dielectrowetting uses a dielectric liquid and non-contacting electrodes. Dielectrowetting works by polarising dipoles within the droplet, can achieve complete wetting, and only introduces a limited contact angle hysteresis^3,10,14^. Past studies have demonstrated the versatility of both electrowetting and dielectrowetting on controlling the contact angle on static or dynamics systems. In 2013, McHale et al. presented a model describing the effect of electric fields on the dynamic contact angle and characterised the rate in which the dynamic contact angle changes with time^14^. McHale’s experiments demonstrated that the contact angle of propylene glycol droplets, subject to alternating current (AC potentials, changes in the few millisecond time scale^14^. In addition, the recent work of Vo and Tran concluded that electrowetting does not affect the initial stages of the contact line dynamics of spreading droplets^15^. In fact, very few studies have addressed the dynamics of fast-moving contact lines, or droplet impact, despite the importance of these conditions in industrial applications, such as inkjet printing or airplane icing^16–18^. Moreover, controlling droplet impact is critical when handling hazardous materials, as impacting and splashing droplets have been proven to be dangerous sources of disease propagation^19,20^. Droplet splashing is also undesirable during the transport of toxic or bio-hazardous liquids, and in medical applications where retaining a sterile environment is critical.

The dynamics of droplet impact are often characterised by the Weber number (We $$= \rho D_0 U_0^2/ \sigma$$, where $$D_0$$ and $$U_0$$ are the droplet diameter and impact velocity, and $$\rho$$ and $$\sigma$$ are the fluid density and surface tension, respectively), the Reynolds number (Re $$= \rho U_0 D_0 / \mu$$, and $$\mu$$ is the liquid dynamic viscosity), and the dimensionless inertial time $$t^* = t\,(U_0/D_0)$$. Several studies have been devoted to predicting the maximum spreading diameter, $$D_{max}$$, of impacting droplets in terms of these parameters, as they can be used to control the coating area of sprays and inkjet systems. Past studies have centred on finding empiric scaling laws of the form of $$D_{max}\propto$$We$$^{\alpha }$$Re$$^{\beta }$$, where $$\alpha$$ and $$\beta$$ are real numbers. For perfectly non-wetting substrates, Eggers et al. (2010) theoretically found a scaling law of the form $$D_{max} \propto$$Re$$^{1/5}f($$We Re$$^{-2/5})$$, which reduces to $$D_{max} \propto$$We$$^{1/2}$$ for droplets impacting at high speeds or to $$D_{max} \propto$$Re$$^{1/5}$$ in a viscous regime^21,22^. Other models have been proposed for dissipating wetting substrates^23,24^. Meirong Song et al. (2017) showed that surfactants increase the spreading diameter of water droplets impacting on to superhydrophobic substrates^25^. More recently, Vo et al. (2020) and Tan et al. (2021), showed that the maximum spreading is augmented by dielectrowetting, while hindering receding and even bouncing^13,26^. The *contact time* of impacting droplets has also been studied extensively. Experimental studies have shown that the contact time of water droplets impacting superhydrophobic surfaces remains constant regardless of the impact velocity^27^. Furthermore, for biological structured surfaces, droplet splitting was found to effectively reduce the contact time^28^.

Abundant studies have centred on the topic of splashing, which is commonly defined as the phenomenon in which a liquid drop disintegrates into droplets following impact on a substrate. Splashing occurs above critical We and Re numbers, when the contact line speed exceeds a critical velocity permitting air entrapment below the advancing droplet front (lamella). Riboux and Gordillo (2014) found that the competition between these aerodynamic and surface tension forces can be effectively described by the splashing parameter, $\beta \approx 3.84 \frac{\mu _g^{1/2} (\rho D_0 U_{0}^5)^{1/6}}{\sigma ^{2/3}}$ (where $\mu _g$ is the air viscosity)^29,30^. In addition, splashing has been shown to depend on surface properties, such as roughness^31^, stiffness^32^, wettability (advancing dynamic contact angle)^33^, the liquid properties, and the ambient pressure^34,35^. Understanding and suppressing splashing is desirable in several applications to avoid cross-contamination. Another undesired consequence of splashing is that splashed micro-droplets can remained suspended in air forming mist that can then transmit diseases^36,37^. Consequently, several studies have been devoted to controlling or suppressing splashing. Past works have demonstrated that splashing can be suppressed by introducing a soft deformable coating to a solid substrate^32^, or by adding surfactants or polymer additives to the liquid forming the droplet^25,38^.

In this work, we study the high-speed impact (high We numbers) of de-ionised water droplets on to a flat superhydrophobic substrate containing interdigated copper electrodes. The electrode array forms a dielectrowetting system that is driven by an alternating current (AC) potential. Our experiments show that the maximum spreading diameter, the contact time, and splashing depend on the dielectrophoretic force acting on the droplet, which, in turn, is controlled by the amplitude of the applied electric potential. In addition, we show that dielectrowetting controls, and even suppresses, droplet bouncing. Finally, we demonstrate that splashing can be dramatically reduced, or even suppressed, by controlling the maximum dynamic contact angle through dieletrowetting.

**Figure 1:**
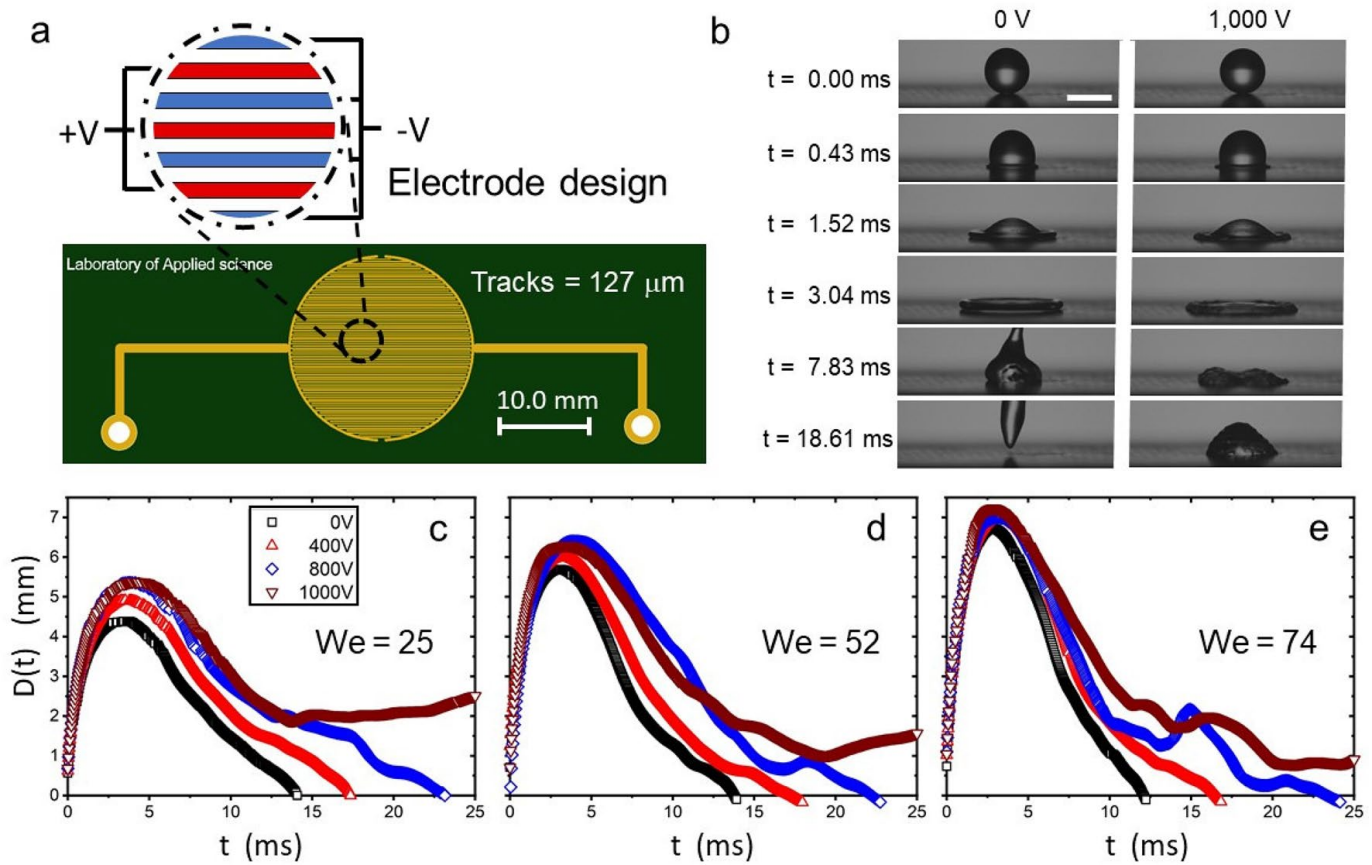
(**a**) Schematics of the PCB substrate showing the electrode configuration and size. (**b**) Image sequence of a water droplet impacting, at $U_0 = 1.01$ m s$^{-1}$ or We = 34, a dielectrowetting flat substrate at 0 V (left) and at a 1000 V (right) electric potentials. The scale bar size is 2.5 mm. Bouncing is observed for the zero potential case but suppressed at 1,000 V. (**d**–**f**) Spreading diameter, in terms of time, at (**c**) We = 25, (**d**) We = 52 and (**e**) We = 74, for various electric potentials.

## Results and discussion

The experiment consists of water droplets impacting a flat Printed Circuit Board (PCB) connected to an AC potential. The tracks of the PCB are organised in a parallel array to create two interdigitated electrodes, as seen in Fig. 1a. The electrodes form a circular overall shape, where tracks have a width and a gap separation of 127 $\upmu$m. The overall diameter of the circular electrode array is 20 mm in diameter (larger than the maximum spreading diameter found within our experimental conditions). The PCB substrates are spray-coated with Glaco to render superhydrophobic behaviour. In our experiments, the dielectrowetting effect is produced at the surface of the interdigitated electrodes by the AC voltage. The peak-to-zero amplitude of the AC voltage, at 1.0 kHz, was varied within the range of 0–1000 V using a high voltage power amplifier (PZD700D, Trek) driven by a function generator set to a pure sine wave (AFG1062, Tektronix). Deionised water was used as the dielectric liquid for all the experiments. Drops were generated by dripping from a 1.0 mm diameter metallic syringe tip connected to a syringe pump (Razel, model R99-E) set at a flow of 1.94 mm$^3$ s$^{-1}$. The characteristic size of the droplet in these experiments is of $2.5 \pm 0.1$ mm and the impact speed was varied in the range of 0.86–2.05 m s$^{-1}$. These parameters covered conditions from smooth spreading to splashing. The impact events were captured by a high-speed camera set to an effective resolution of 14.05 $\upmu$m per pixel at 23,000 frames per second. The geometry and the electrical connections of the PCB substrate prevented us from visualising the impact events from various directions. Consequently, the field of view of our imaging system is parallel to the length of the electrodes. No preferred asymmetry was observed on the impacting experiments.

**Figure 2:**
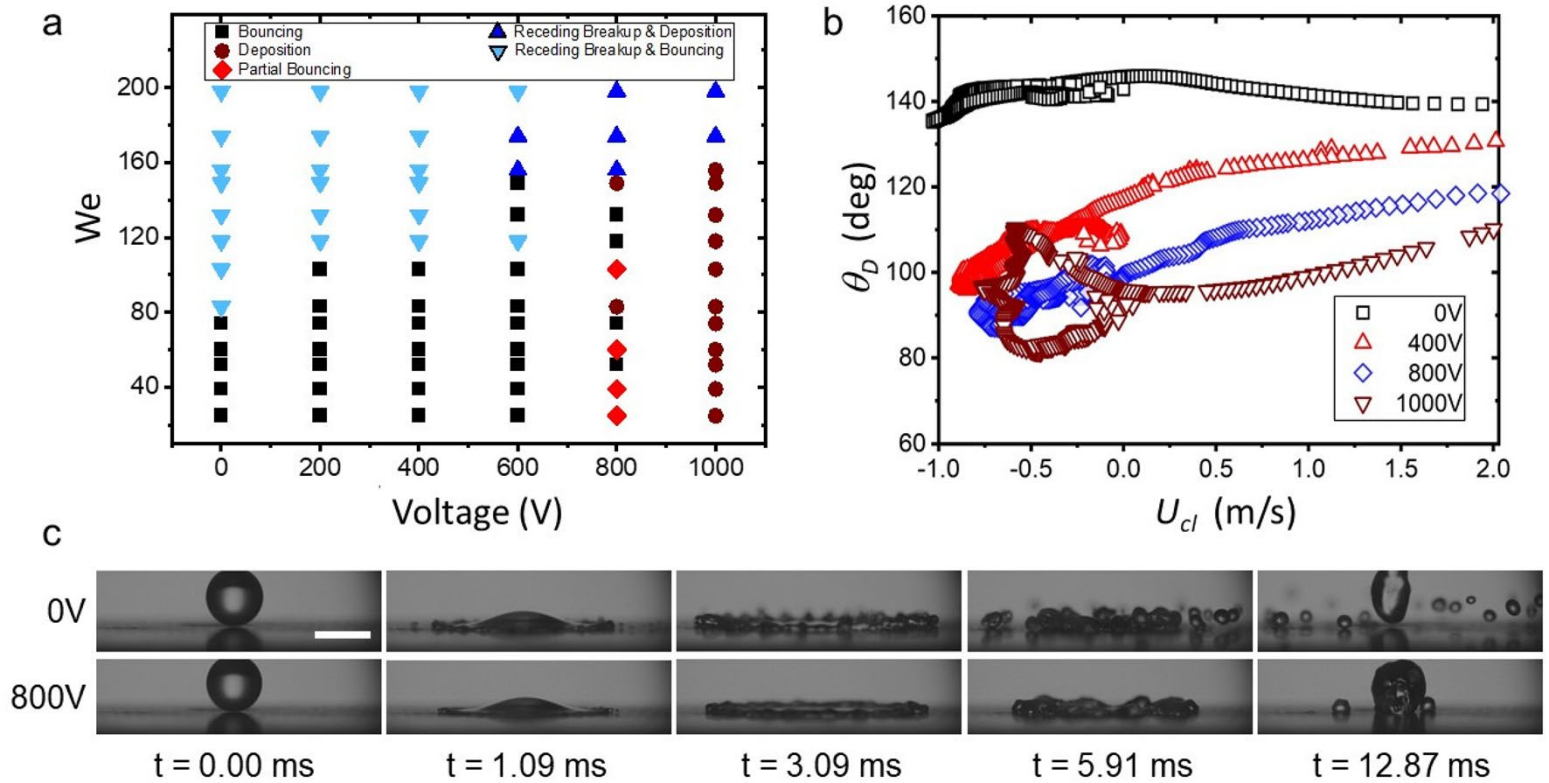
(**a**) Bouncing/no-bouncing map behaviour in terms of the Weber number and the applied AC voltage. (**b**) Dynamic contact angle in terms of the contact line velocity for different applied dielectrowetting voltages at We = 52. (**c**) Snapshots of a water droplet impacting at We = 74; at $V=0$ V receding breakup and bouncing is observed but receding breakup and deposition is observed at $V=800$ V (the droplet remains on the substrate). Scale size = 2.5 mm.

**Figure 3:**
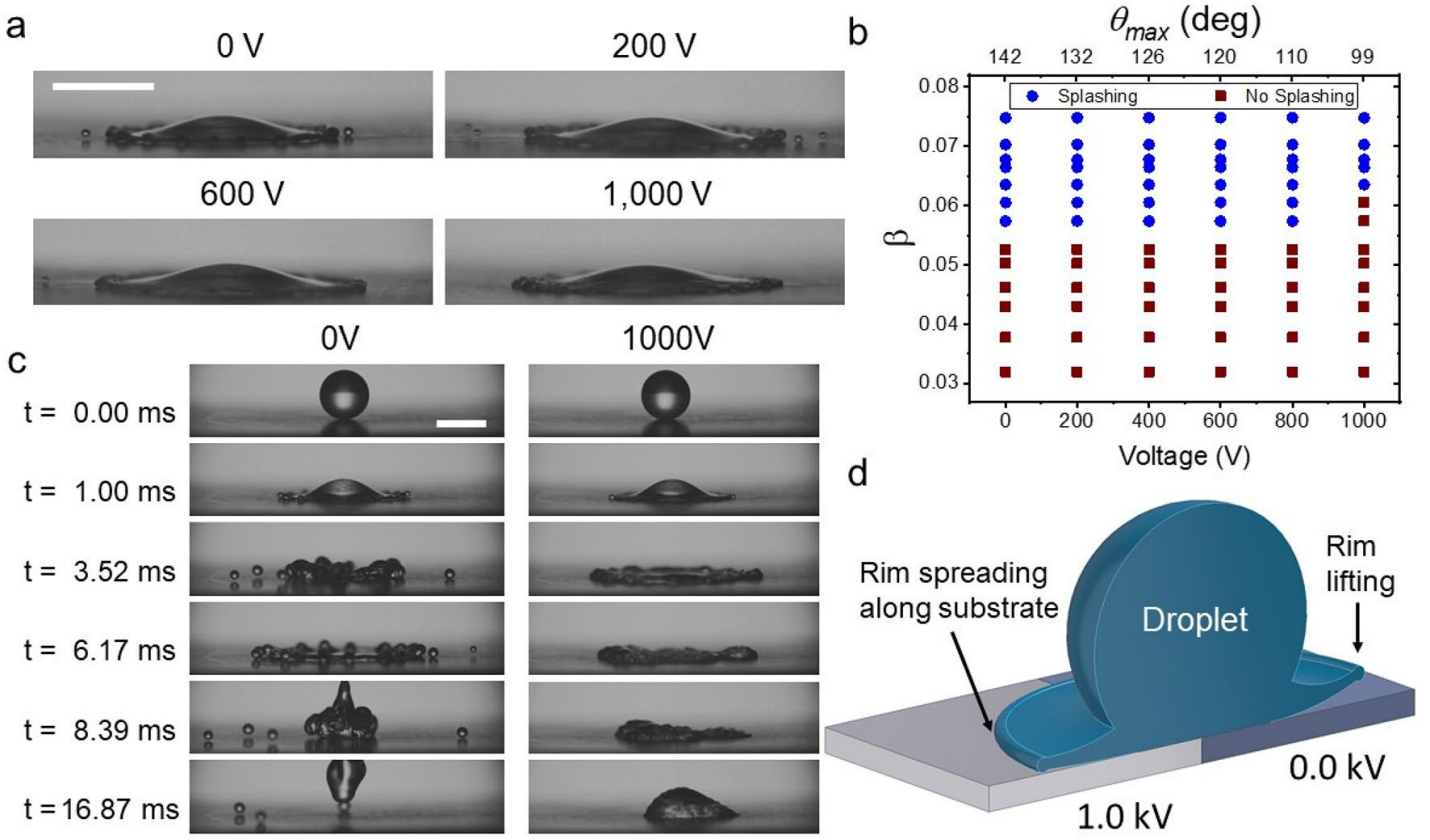
(**a**) Droplet snapshots, at We $= 104$ and $t = 2.69$ ms after impact. This time corresponds to the onset of splashing for 0 V but the behaviour is changed by the dielectrowetting potential. (**b**) Splashing/no-splashing map in terms of the splashing parameter $\beta$ and the dielectrowetting voltage or the dynamic contact angle. (**c**) A water droplet impacting, at $\beta = 0.061$ ($We = 118$), dielectrowetting substrates at two potentials. Splashing is observed at 0 V but no splashing is observed at 1000 V. (**d**) Schematic of the rim behaviour: at 0 V the rim detaches and flies off the surface promoting splashing. In contrast, a dielectrophoretic potential of 1000 V causes the rim to spread over the surface inhibiting splashing. Scale size = 2.5 mm.

Our experimental conditions covered the range of 20 < We < 200 which, thereby by using superhydrophobic substrates and dielectrowetting, included impact events ranging from deposition to bouncing. Under these conditions, we identified six characteristic behaviours within this range of Weber numbers, namely *(smooth) deposition, (smooth) bouncing, receding breakup and deposition, receding breakup and bouncing, partial bouncing, and splashing.* In *(smooth) deposition* an impacting droplet spreads over the substrate to achieve a maximum spreading diameter to then recede to an equilibrium diameter (Fig. 1b (right)). Under these dynamics, the droplet always remains attached to the substrate. Under conditions of *(smooth) bouncing* the droplet impacts, advances to the maximum spreading diameter, then rapidly recedes, and its entire volume bounces off the substrate, as seen in Fig. 1b (left). In *receding breakup and deposition* the droplet spreads but its rim (lamella) detaches from the substrate and breaks up into smaller droplets during receding. After breakup, the rest of the droplet remains attached to the substrate (Fig. 2c bottom). In contrast, during *receding breakup & bouncing*, the droplet rim breaks up and the remaining droplet volume bounces off away from the substrate (Fig. 2c top). In *partial bouncing* the droplet rim recedes without breaking up, but the droplet volume is split by the recoil; a fraction of the droplet stays attached to the substrate but the rest bounces off. During *splashing*, the droplet rim detaches from the substrate and fragments during spreading as seen in Fig. 3c (left).

In our experiments, we parametrically varied both the speed of impact (to adjust the We number) and the amplitude of the electric potential (to modify the strength of the dielectrophoretic force). The impact of droplets was recorded by the high-speed camera and then analysed by image analysis to obtain both the spreading diameter and the dynamic contact angle in terms of the time from impact. The results demonstrate that the contact line dynamics are influenced by the strength of the dielectrowetting potential, as seen in Fig. 1c–e. In our experiments, the maximum spreading diameter increases by increasing the applied voltage, while the contact time considerably increases to the point where bouncing is suppressed altogether for applied voltages of 1000 V. This behaviour is well-captured by Fig. 1b, where the bouncing of a droplet, otherwise found at 0 V, is suppressed by a dielectrowetting potential set at 1000 V. According to the Young–Lipmann equation, a dielectrophoretic force leads to a reduction of the contact angle, thus lowering the receding speed of the contact line and suppressing bouncing^13^.

Figure 2a shows the bouncing/no-bouncing regime map in terms of the Weber number and the applied voltage. As seen, without an electric potential (0 V), only two conditions are observed: (smooth) bouncing, and receding breakup and bouncing. In contrast, dramatic differences arise for dielectrowetting, e.g. bouncing is suppressed across all Weber numbers with an applied potential of 1000 V. In fact, a dielectrowetting potential of 1000 V also suppresses receding breakup resulting in (smooth) deposition at all conditions for We < 170 (Fig. 2a). Other examples, shown in Fig. 2c, see receding breakup and bouncing, at 0 V and We = 74, being replaced by conditions where breakup is considerably reduced and bouncing suppressed at a potential of 800 V. The effect of dielectrowetting is also best observed at the critical splashing velocity, being $U_0 = 1.75$ m s$^{-1}$ for our water droplets (Fig. 3a). Accordingly, the onset of splashing, at 0 V, occurs 2.69 ms after impact. In contrast, at this time, splashing is seen reduced at dielectrowetting potentials > 200 V, to the point where splashing is entirely suppressed, together with receding breakup and bouncing, at a potential of 1000 V (Fig. 3c).

### Splashing

The reduction or suppression of splashing and receding breakup is explained by a series of factors triggered by the electric potential.

- Firstly, the electric field reduces the receding velocity affecting the growth of instabilities. As discussed by other works, receding breakup arises from Kelvin–Helmholtz instabilities of wave number $k \sim u^2_r/ 3\sigma$. Therefore, reducing the receding speed, $u_r$, decreases *k* which, in turn, reduces the number of breakup points at the rim and the number of breakup droplets^25^. This is evident in Fig. 1c–e.
- Secondly, the dynamic wettability of a substrate controls splashing and for a given liquid, large advancing contact angles favour splashing^31,33,39^. Our experiments show that the dielectrophoretic force affects the contact line dynamics by decreasing the maximum advancing contact angle. This effect is evident from Fig. 2b where the dynamic contact angle, at a contact line speed of $U_{cl}=$ 2.0 m s${^{-1}}$, varies from $\theta _{D}=$ 140$^{\circ }$ for 0 V, to $\theta _{D}=$ 110$^{\circ }$ for 1000 V (Fig. 2b).
- Third, as argued elsewhere, the upward force induced by air entrapment is suppressed by wetting transitions^25,40^. Quintero et al. (2019) showed that the rim of a spreading droplet is not in contact with a superhydrophobic surface and that wettability determines the gas dynamics of the trapped air beneath the drop^41^. Here, we argue that dielectrophoretic forces change wettability, registered as a change of contact angle, driving the rim to contact the substrate hindering splashing; as illustrated by Fig. 3d.
- Finally, electric fields are also known to produce a stabilising effect on capillary instabilities, which in our conditions would inhibit splashing and receding breakup^42,43^.

As described in the introduction, splashing is an undesired phenomenon affecting multiple industrial processes such as spray coating and painting. Recent studies have concluded that splashing is determined by the liquid and the substrate properties, and the impacting conditions. The impacting and the liquid properties have successfully parametrised splashing through the splashing parameter^29^ and the substrate properties (through the maximum advancing contact angle)^33^. In brief, $\beta$, the splashing parameter, defines the splashing threshold condition for impacting droplets as a competition between the rim’s (lamella) lifting force, the surrounding gas viscosity and the capillary retraction (surface tension)^29^. The maximum advancing contact angle, $\theta _{max}$, is the angle formed by the droplet’s rim during the first milliseconds of spreading, when this angle remains constant^33^. In a previous work, we demonstrated that $\theta _{max}$ and the splashing parameter, $\beta$, are sufficient to determine the splashing/no-splashing threshold for smooth flat substrates^33^. The splashing parameter, combined with the mean and the peak-to-peak heights of the features within a substrate, has also been shown to determine the splashing dynamics on rough surfaces^31^.

**Figure 4:**
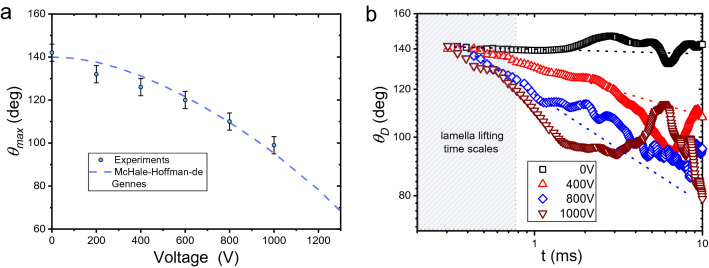
(**a**) Maximum advancing contact angle in terms of the electric potential strength; the McHale–Hoffman–de Gennes model with a Voltage threshold of $V_{TH} =1600$ V is seen as a dashed lines. (**b**) Dynamic contact angle in terms of time for various potential strengths at We = 52; dashed lines show exponential fits for early times (t < 1.0 ms).

The experiments discussed here, with electric fields, demonstrate that splashing/no-splashing regimes are readily controlled by the splashing parameter and the electric potential, in Fig. 3b. As seen, without the electric field applied, the splashing parameter threshold is found at $\beta =$ 0.055, which is consistent with the critical splashing parameter expected for superhydrophobic materials, with $\theta _{max} > 145^{\circ }$^33^. In fact, our past results also indicate that, at $\beta =$ 0.055, any action that reduces the value of the maximum dynamic contact angle to, or below, 130$^{\circ }$, should suppress splashing. Interestingly, Fig. 4a shows a steady reduction of the contact angle by an increasing dielectrowetting potential; this behaviour is well in agreement with the McHale-Hoffman-de Gennes model^14^ and consistent with the work of Blake et al. (2000) concluding that the dynamic contact angle is reduced at increasing electrostatic fields^44^. Regardless of this steady reduction on the contact angle, splashing is not suppressed except for the highest electrical field. In the dielectrowetting experiments, we observe a reduction of the contact angle to values well below the predicted threshold ($\theta _{max}= 130^{\circ }$), to the point where $\theta _{max}= 110.0 \pm 4.0^{\circ }$ and splashing is not suppressed. Splashing is only suppressed at a dielectrowetting potential of 1000 V or at a $\theta _{max} = 99.0 \pm 4.0^{\circ }$.

This phenomenon is explained by the speed of action of the dielectrophoretic force. A closer look at Figs. 2c and 3c reveals that, without a dielectrowetting potential, splashing occurs within the first 1.1 ms after impact; with the lifting of the lamella typically occurring at the first 0.4–1.1 ms window after impact (as seen in the supplemental material of Quetzeri-Santiago et al.^33^). These time scales are too short for the action of the dielectrophoretic force to affect the splashing dynamics. Indeed, as observed in Fig. 1c–e, the action of the dielectrophoretic force on the spreading dynamics is only visible after the first $\sim$ 2.0 ms. Our results and analysis (Fig. 4b), demonstrate that, as described by McHale et al. (2013), at early times, the dynamic contact angle varies exponentially with time at a rate that only sees differences of tenths of degrees after 1.0 ms^14^; these observations being consistent with the work by Vo and Tran (2021) where dielectrophoretic effects take up $\sim 1.0$ ms to arise^13^. Furthermore, as seen in Fig. 4b, at a potential of 400 V, a droplet needs to spread for 2.0 ms to achieve its maximum spreading contact angle ($\theta _{max} = 126^{\circ }$). Summarising, low-potential dielectrophoresis effects are about one or two milliseconds too slow to modify the wettability of the substrate enough to stop splashing. We conclude that the speed of dielectrowetting effects on substrate wettability is fast enough to affect droplet bouncing, but, at low electrical fields, is too slow to affect the splashing dynamics of water droplets.

## Conclusion

In this work, we have demonstrated that dielectrowetting is an effective solution to prevent bouncing and receding breakup, and, at high electrical fields, can suppress splashing upon drop impact. We also found that the speed in which the advancing contact angle changes depends on the potential strength and needs to occur at scale times relevant to the lifting of the lamella to affect splashing. Controlling bouncing and splashing are necessary to avoid, for instance, the spreading of diseases, toxins and germs. For example, controlling the generation of unwanted droplets, as the result of splashing, is critical in medical environments, screening and testing, surgery, chemistry, material processing and fluid formulation, in order to prevent cross-contamination. Most of the previous research focused on suppressing splashing has concentrated on changing the liquid properties or permanently modifying the substrate chemistry or properties to avoid splashing. However, in most situations, this is not desirable, or even practical. Consequently, dielectrowetting could be a possible solution in these environments where the field could be controlled on demand to curb droplet rebound and/or splashing.

## Methods

### Sample fabrication and operation

The PCB substrate was manufactured by JLCPCB (JiaLiChuang Co Limited, Hong Kong) with a substrate thickness of 1.6 mm and an Electroless Nickel Immersion Gold finish (ENIG-RoHS). The PCB has copper tracks with a standard 0.04 mm thickness. The overall diameter of the circular electrode array is 20.0 mm in diameter (larger than the maximum spreading diameter found within our experimental conditions). SU-8 photoresists (SU-8 2 from Kayaku-MICROCHEM) was uniformly applied to the substrate with a palette knife to fill the $\approx 40$ $\upmu$m gaps found between the electrode tracks. Based on the procedure found in^14^, the coated substrate was firstly baked for 1 min at 65 Celsius, then cured for 15 min in a UV crosslinker (UVP Analytikjena, 254 nm), and then hard baked for 10 min at 155 Celsius. After curing and baking, excess photoresists was sanded off by progressively finer grits, i.e. 600-grit, 800-grit and 1000-grit flat sandpapers. The surface was then hand polished with a regular grade acrylic compound (G3, Farécla). In the final step, Glaco was applied, as a spray, to make the surface superhydrophobic and prevent pinning to the surface.

The PCB substrate was tested at the following frequencies: 0.1, 1.0 and 10.0 kHz, at 800 V. At 0.1 kHz a large contact angle hysteresis and droplet boiling were found. In contrast, at 1.0 and 10.0 kHz, contact angle hysteresis was low and we found no significant differences in the dynamics of the contact angle between these two frequencies. At 1.0 kHz the dielectrowetting effect is stable and reliable; however, we found that at 10.0 kHz the substrate short-circuited after long continuous operation. In addition, the electrical breakdown of the substrate is found at around 1200 V.

### Shadowgraphy details

The impact events were captured by a Phantom V710 high-speed camera coupled to a 12 $\times$ Navitar microscope lens in a shadowgraph configuration. The camera resolution was set to $1280 \times 256$ pixels$^2$ with a sample rate of 23, 000 frames per second with an exposure time of 10 $\upmu$s. The effective resolution of all the experiments was of 14.05 $\upmu$m per pixel. The camera was inclined $\approx$ 2$^{\circ }$ to obtain a clear image of the contact line; the effect of this inclination on the measurement of the contact angle is negligible^31^. A 300 W LED light source coupled to an optical diffuser was utilised to generate a uniform bright background.

### Measurement of the dynamic contact angle

Image analysis was performed on spreading experiments to extract dynamic contact angles $\theta _D$, spreading diameters, impact speeds and droplet sizes by a custom MATLAB code. Details of this Matlab algorithm are found elsewhere^45^.
